# Myeloid sarcoma: more and less than a distinct entity

**DOI:** 10.1007/s00277-023-05288-1

**Published:** 2023-06-07

**Authors:** Giuseppe G. Loscocco, Alessandro M. Vannucchi

**Affiliations:** 1 Department of Experimental and Clinical Medicine, CRIMM, Center for Research and Innovation of Myeloproliferative Neoplasms, Azienda Ospedaliero-Universitaria Careggi, University of Florence, Florence, Italy; 2 Doctorate School GenOMec, University of Siena, Siena, Italy

**Keywords:** Myeloid sarcoma, Granulocytic sarcoma, Chloroma, Extramedullary leukaemia, Myeloid neoplasms

## Abstract

Myeloid sarcoma (MS) is a distinct entity among myeloid neoplasms defined as a tumour mass of myeloid blasts occurring at an anatomical site other than the bone marrow, in most cases concomitant with acute myeloid leukaemia (AML), rarely without bone marrow involvement. MS may also represent the blast phase of chronic myeloproliferative neoplasms (MPN) and myelodysplastic syndromes (MDS). However, the clinical and molecular heterogeneity of AML, as highlighted by the 2022 World Health Organization (WHO) and International Consensus (ICC) classifications, indirectly define MS more as a set of heterogeneous and proteiform diseases, rather than a homogeneous single entity. Diagnosis is challenging and relies mainly on histopathology, immunohistochemistry, and imaging. Molecular and cytogenetic analysis of MS tissue, particularly in isolated cases, should be performed to refine the diagnosis, and thus assign prognosis guiding treatment decisions. If feasible, systemic therapies used in AML remission induction should be employed, even in isolated MS. Role and type of consolidation therapy are not univocally acknowledged, and systemic therapies, radiotherapy, or allogeneic hematopoietic stem cell transplantation (allo-HSCT) should be considered. In the present review, we discuss recent information on MS, focusing on diagnosis, molecular findings, and treatments also considering targetable mutations by recently approved AML drugs.

## Introduction

Myeloid neoplasms, typically liquid tumours, may manifest as extramedullary masses, representing a significant diagnostic and therapeutic challenge [1]. In 1811, more than two centuries ago, extramedullary manifestation of acute myeloid leukaemia (AML) was firstly described [2] and named “chloroma”; this term derived from χλωρός (“chloros”), the Greek word for “green,” based on the tumour’s appearance related to the oxidation of myeloperoxidase (MPO) within the granules of immature myeloid cells [3, 4]. Lately, in 1965, the word “myeloblastoma” was coined, encompassing a more appropriate histologic description of the lesion [5]. Concurrently, the terms “granulocytic sarcoma” [6, 7] and “myeloid sarcoma” (MS) [8] were proposed. MS appears to be the most appropriate term for this entity, given its adoption by the World Health Organization (WHO) in the classification of myeloid neoplasms originally in 2008 [9] and then in revised version of 2016 [10]. In the latter, MS is defined as a tumour mass of myeloid blasts, with or without maturation, occurring at an anatomical site other than the bone marrow, specifying the mandatory effacement of local tissue architecture to properly define MS [10]. The latest WHO [11] and International Consensus Classification (ICC) [12] published in 2022 maintained the disease definition of MS. In Table 1, the latest ICC and WHO classifications for AML, including MS entity, are summarized.

**Table 1:** Latest classifications of acute myeloid leukemia

2022 International consensus classification (ICC) [12]	2022 5th-WHO classification [11]
-Acute promyelocytic leukemia (APL) with t(15;17)(q24.1;q21.2)/*PML::RARA* (blasts ≥10%) -APL with other *RARA* rearrangements^*^ (blasts ≥10%)	Acute promyelocytic leukaemia with *PML::RARA* fusion (no blasts cutoff)
AML with t(8;21)(q22;q22.1)/*RUNX1::RUNX1T1* (blasts ≥10%)	Acute myeloid leukaemia with *RUNX1::RUNX1T1* fusion (no blasts cutoff)
AML with inv(16)(p13.1q22) or t(16;16)(p13.1;q22)/*CBFB::MYH11* (blasts ≥10%)	Acute myeloid leukaemia with *CBFB::MYH11* fusion (no blasts cutoff)
AML with t(6;9)(p22.3;q34.1)/*DEK::NUP214* (blasts ≥10%)	Acute myeloid leukaemia with *DEK::NUP214* fusion (no blast cut-off)
-AML with t(9;11)(p21.3;q23.3)/*MLLT3::KMT2A* (blasts ≥10%) -AML with other *KMT2A* rearrangements^**^ (blasts ≥10%)	Acute myeloid leukaemia with *KMT2A* rearrangements (no blasts cutoff)
-AML with inv(3)(q21.3q26.2) or t(3;3)(q21.3;q26.2)/*GATA2::MECOM(EVI1)* (blasts ≥10%) -AML with other *MECOM* rearrangements^***^ (blasts ≥10%)	Acute myeloid leukaemia with *MECOM* rearrangements (no blasts cut-off)
-AML with other rare recurring translocations (including *NUP98* rearrangement and *RBM15::MRTF1* fusion) (blasts ≥10%)	Acute myeloid leukaemia with other defined genetic alterations (no blasts cut-off)
AML with t(9;22)(q34.1;q11.2)/*BCR::ABL1* (blasts ≥20%)	Acute myeloid leukaemia with *BCR::ABL1* fusion (blasts ≥20%)
AML with mutated *NPM1* (blasts ≥10%)	Acute myeloid leukaemia with *NPM1* mutation (no blasts cut-off)
AML with in-frame bZIP *CEBPA* mutations (blasts ≥10%)	Acute myeloid leukaemia with *CEBPA* mutation (blasts ≥20%)
-AML with mutated *TP53* (blasts ≥20%)	Acute myeloid leukaemia, myelodysplasia-related (blasts ≥20%)
-AML with myelodysplasia-related gene mutations (blasts ≥20%) Defined by mutations in *ASXL1, BCOR, EZH2, RUNX1, SF3B1, SRSF2, STAG2, U2AF1, or ZRSR2*	Acute myeloid leukaemia, myelodysplasia-related (blasts ≥20%) Defined by mutations in *ASXL1*, *BCOR*, *EZH2*, *RUNX1*, *SF3B1*, *SRSF2*, *STAG2*, *U2AF1*, or *ZRSR2*
AML with myelodysplasia-related cytogenetic abnormalities (blasts ≥20%). Defined by detecting a complex karyotype ( ≥3 unrelated clonal chromosomal abnormalities in the absence of other class-defining recurring genetic abnormalities), del(5q)/t(5q)/add(5q), -7/del(7q), +8, del(12p)/t(12p)/add(12p), i(17q), -17/add(17p) or del(17p), del(20q), and/or idic(X)(q13) clonal abnormalities	Acute myeloid leukaemia, myelodysplasia-related (blasts ≥20%). Defined by a complex karyotype (≥3 abnormalities); 5q deletion or loss of 5q due to unbalanced translocation; monosomy 7, 7q deletion, or loss of 7q due to unbalanced translocation; 11q deletion; 12p deletion or loss of 12p due to unbalanced translocation; monosomy 13 or 13q deletion; 17p deletion or loss of 17p due to unbalanced translocation; isochromosome 17q; idic(X)(q13)
AML not otherwise specified (NOS) (blasts ≥20%)	AML, defined by differentiation (blasts ≥20%)^#^
Myeloid sarcoma	Myeloid sarcoma

^*^Includes AMLs with t(1;17)(q42.3;q21.2)/*IRF2BP2*::*RARA*; t(5;17)(q35.1;q21.2)/*NPM1*::*RARA*; t(11;17)(q23.2;q21.2)/*ZBTB16*::*RARA*; cryptic inv(17q) or del(17)(q21.2q21.2)/*STAT5B*::*RARA*, *STAT3*::*RARA*; other genes rarely rearranged with *RARA*:*TBL1XR1* (3q26.3), *FIP1L1* (4q12), *BCOR* (Xp11.4)

^**^Includes AMLs with t(4;11)(q21.3;q23.3)/*AFF1*::*KMT2A#*; t(6;11)(q27;q23.3)/*AFDN*::*KMT2A*; t(10;11)(p12.3;q23.3)/*MLLT10*::*KMT2A*; t(10;11)(q21.3;q23.3)/*TET1*::*KMT2A*; t(11;19)(q23.3;p13.1)/*KMT2A*::*ELL*; t(11;19)(q23.3;p13.3)/*KMT2A*::*MLLT1#* (# Occurs predominantly in infants and children)

^***^Includes AMLs with t(2;3)(p11~23;q26.2)/*MECOM*::?; t(3;8)(q26.2;q24.2)/*MYC*, *MECOM*; t(3;12)(q26.2;p13.2)/*ETV6*::*MECOM*; t(3;21)(q26.2;q22.1)/*MECOM*::*RUNX1*

^*#*^Includes AML with minimal differentiation, AML without maturation, AML with maturation, acute basophilic leukaemia, acute myelomonocytic leukaemia, acute monocytic leukaemia, acute erythroid leukaemia, and acute megakaryoblastic leukaemia

MS can occur in the context of intramedullary AML (synchronous), but may also occur in an isolated form with an essentially normal bone marrow which is usually followed by the development of metachronous AML [13]. MS may represent a form of blast transformation in patients with myeloproliferative neoplasms (MPN) [14, 15], myelodysplastic syndromes (MDS) [16], or MDS/MPN [17, 18]. Moreover, MS may be the first clinical manifestation of AML relapse, particularly after allogeneic hematopoietic stem cell transplantation (allo-HSCT) [19].

Epidemiology of MS is difficult to assess, also owing to the multitude of terms employed to describe the entity as well as the fact that in most studies, the incidence was reliant upon findings derived from descriptions of lesions without histologic confirmation. One of the oldest study (collecting patients diagnosed from 1949 to 1969 in Japan) documented an incidence of 8% of MS on autopsy from patients dying by AML [20].

Subsequently, larger studies documented that the rates of synchronous and isolated MS at diagnosis ranged from 0.2 to 2.8% and 0.6 to 0.8%, respectively [21–25].

There are scanty information about the rate of MS, either isolated or synchronous, at relapse after achieving complete remission (CR) with chemotherapy in patients with de novo AML; conversely, more data are available in the setting of post allo-HSCT, in which the incidence of MS was reported at 5–12% accounting for 7–46% of total relapses; of note, approximately 70% of MS after allo-HSCT are isolated [19, 26–29]. Considering MS following allo-HSCT as relapse, no significant correlations between conditioning intensity, graft source, or the presence/absence of acute and chronic graft-versus-host disease (GVHD) were reported [30, 31]. Specifically, the risk of post allo-HSCT MS relapse in patients with a previous MS diagnosis is not well established and reported data are conflicting [30, 32].

MS has a slight male predominance and may occur at any age and at any site of the body. Organs most commonly involved include the skin (defined as leukaemia cutis), lymph nodes, genitals, breast, gastrointestinal (GI) tract, peritoneum, bone, and central nervous system (CNS) [13, 33–35]. In the largest study from US registry, a total of 94,185 cases of AML were reported from 2004 to 2013 of whom 746 patients were diagnosed with MS (0.8%). The median age was 59 years, and 56.1% were male. In that study, the three most common sites of presentation were connective/soft tissues (31.3%), skin/breast (12.3%), and GI tract (10.3%)[25]. Moreover, the frequency of the sites of organ involvement by MS was comparable between synchronous and isolated MS [13, 25].

## Biological background and pathogenesis

The mechanisms for MS to develop are largely unknown. Some studies have focused on cell-cell and cell-matrix interactions within the bone marrow microenvironment analysing adhesion molecules and chemokine receptors/ligand interactions [36]. Differential expression of cellular adhesion molecules was initially reported, with leukaemia cells in patients with MS more frequently expressing CD56 (also known as neural cell adhesion molecule, NCAM) [37–39]. Homophilic binding mediated by CD56 was hypothesized to promote the binding of leukemic blasts to tissues expressing CD56 including adipose/soft tissue, skeletal muscle, GI tract, testicular, and brain, known frequent localization of MS [40]. However, subsequent studies failed to confirm overexpression of CD56 in most cases; moreover, the rate of CD56-positive leukemic cells was similar in patients with and without MS [24, 41].

Another surface protein, electively expressed on mononuclear cells, speculated to be related with MS development, is CD11b (surface β2-integrin member macrophage-1 antigen) [42]. However, these findings more reflect the enrichment of MS blasts with monoblastic or myelomonocytic phenotype and CD11b expression, rather than a direct causality [24, 43].

Chemokine receptor/ligand interactions orchestrate the migration of leukemic cells to peripheral tissues. In this regard, 15 paediatric AML patients with leukaemia cutis and 10 AML patients without skin involvement were studied. Interestingly, compared to controls, blood leukemic blasts significantly overexpressed CCR2. Moreover, leukaemia cutis cells displayed a different set of receptors (CCR5, CXCR4, CXCR7, and CX3CR1) [44]; interactions with epithelial CXCL12, a ligand for both CXCR4 and CXCR7, may contribute to the development of MS [45].

More recently, Yang et al. [46], employing single-cell RNA sequencing on BM and MS (leukaemia-cutis) samples, were able to detect a complement C1Q+ macrophage-like leukaemia subset, which was enriched within MS and pre-existed in BM. The authors demonstrated that C1Q expression, which was modulated by transcription factor MAF BZIP transcription factor B, endowed leukaemia cells with tissue infiltration ability; on the other hand, leukaemia cell dissemination was sustained by tissue fibroblasts that attract C1Q+ leukaemia cells via C1Q− globular C1Q receptor recognition and stimulation of transforming growth factor β1 synthesis. Moreover, univariate and multivariate analyses demonstrated adverse prognosis significance of C1Q expression in large cohort of AML patients [46].

Other factors that may contribute to MS development, particularly the subset of post allo-HSCT relapse, could be related to escape from immune surveillance; however, solid data are lacking. In two independent studies, the overexpression of the immune checkpoints programmed cell death protein 1 (PD-1) or programmed death-ligand 1 (PD-L1) was documented in only 7–10% of MS cases [47, 48].

## Diagnosis: imaging and histopathology

Histopathological diagnosis of MS can be challenging, especially when MS occurs as isolated manifestation. It can be misdiagnosed as a malignant lymphoproliferative disorder, including Hodgkin lymphoma, histiocytic lymphoma, mucosa-associated lymphoid tissue lymphoma, large-cell lymphoma, Ewing sarcoma, thymoma, round blue cell tumours, poorly differentiated carcinoma, or other rare hematopoietic neoplasm as blastic plasmacytoid dendritic cell neoplasm [13, 37, 49, 50]. Of note, MS must be distinguished from non-effacing extramedullary blastic proliferations as well as extramedullary haematopoiesis following administration of growth factors, particularly granulocyte colony-stimulating factor (G-CSF) that can produce pseudotumoral masses [51]. Moreover, accumulation of mature hematopoietic cells can occur in advanced stage of MPN, particularly in myelofibrosis, as a manifestation of extramedullary haematopoiesis pathogenetically associated with the derangement of bone marrow microenvironment [51]. It may be challenging to distinguish these entities; in general, lack of a significant blast component supports a diagnosis of extramedullary haematopoiesis, excluding MS [51]. Historical retrospective series by Meis et al. reported a misdiagnosis rate of 75% [52], which was much lower in more recent series (from 25 to 47%) [53–55]. In the latter, misdiagnosis most commonly occurred due to inadequate immunophenotyping of MS lesion and it was not corrected until a diagnosis of AML was later established by bone marrow biopsy or peripheral blood smear [53–55]. Of note, in patients where lesion is in unusual sites and the risk of biopsy is high, treatment often starts without histopathology diagnosis in some patients with known AML (and synchronous MS). In these cases, the regression of lesions following leukaemia-directed therapy suggests the diagnosis of a MS *ex adiuvantibus*. Fine needle aspiration is usually inadequate to confirm the diagnosis; therefore, biopsy in patients without AML is mandatory. In selected case, radiologically guided core biopsy of the tumour mass may represent a valid alternative [56, 57]. However, the diagnosis of isolated MS warrants a bone marrow evaluation, including immunophenotyping, cytogenetic, and molecular analysis, to exclude the possibility of a concomitant AML.

The best imaging modality depends on the anatomic sites; magnetic resonance imaging (MRI) is more sensitive to assess for central nervous system (CNS), spinal, and musculoskeletal lesions, whereas CT scan is best suited for soft tissue assessments [58–61]. 18-Fluorodeoxy-glucose positron emission tomography/CT (18-FDG-PET/CT) can be performed to search for multiple site involvement, and it is usually used for planning radiotherapy and monitoring the treatment response [62, 63].

Morphologic appearance of MS by haematoxylin-eosin staining varies according to the degrees of differentiation of myeloblasts which may have features of promyelocytic or granulocytic maturation (in these cases, the presence of eosinophilic precursors is a helpful indicator of MS), whereas in most cases, they show a myelomonocytic or a pure monoblastic morphology [51, 64]. Predominance of erythroblasts or megakaryoblasts in the context of MS is extremely rare and is often reported in cases of blastic transformation of MPN or MDS/MPN[13]. Fresh tissue is usually not available; thus, the diagnosis is confirmed by using immunohistochemistry on formalin-fixed, paraffin-embedded (FFPE) biopsies. In this regard, an extensive antibody panel should be performed. In Table 2, the rates of positive immunohistochemical staining according to the largest studies are reported. Overall, CD68/KP1 positive staining is documented in more than 90% of MS samples, whereas CD68/PGM1, which has a greater specificity for monocytes and macrophages, stains positive in about 50% of cases [13, 65, 66]. CD33, CD43, CD117, HLA-DR, and myeloperoxidase (MPO) are variably expressed in 40–95% of cases [13, 65, 66]; CD34 is positive in approximately 30% of cases, typically in more immature myeloid cells, whereas it is absent typically in promyelocytic and monoblastic variants [13, 65, 67]. Other studies reported positivity for CD99 [13], CD56 [13, 65–67], terminal-deoxy-nucleotidyl-transferase (TdT) [13, 66], CD163 [66], CD123 [66], CD4 [13, 66, 67], CD14 [66], and CD30[13]. Exceptionally, aberrant expression of cytokeratins as AE1/AE3 and CK8/18 was reported [68]. Some recent reports reported a not-infrequent BCL-2 positivity [69, 70]. Erythroid cases are positive for glycophorin A/C and CD71[71, 72], whereas megakaryoblastic MS express CD61 and von Willebrand factor (vWF) [73, 74].

**Table 2:** Immunohistochemistry in myeloid sarcoma

Immunohistochemical stains	Rates of positivity (%)	References
CD68/KP1	90–100	[13, 65, 66]
CD33	55–94	[66, 67]
CD43	9–100	[66, 67]
MPO	50–88	[13, 65, 66]
Lysozyme	23–92	[66, 67]
HLA-DR	41–86	[13, 65, 67]
CD117	55–80	[13, 67]
CD99	56	[13]
CD68/PG-M1	51–53	[13, 65]
CD34	27–44	[13, 65, 67]
CD56	5–30	[13, 65–67]
TdT	0–32	[13, 66]
CD163	30	[66]
CD123	23	[66]
CD4	1–22	[13, 66, 67]
CD14	13	[66]
CD30	2	[13]
BCL-2	80	[69, 70]

## Cytogenetic and molecular characteristics

According to available reports, MS cells demonstrate clonal cytogenetic abnormalities in 54–70% of cases [13, 67]. Although some older studies supported a higher than expected proportion of patients with (8;21)(q22;q22.1)/*RUNX1*::*RUNX1T1* translocation [37, 75–78], subsequent larger studies demonstrated that *RUNX1*::*RUNX1T1* is a rare event (2–3% of cases) in MS [13, 24]. Moreover, inv(16)(p13.1q22)/*CBFB*::*MYH11* translocation was associated with MS in two series, particularly at abdominal sites [79–82]. Others reported cytogenetic alterations in MS to include t(9;11), del(16q), t(8;17), t(8;16), and t(1;11) and chromosome 4, 7, 8, or 11 abnormalities [13, 83, 84]. However, no clear association between cytogenetics of intramedullary AML and the occurrence of extramedullary disease can be demonstrated. The mutational landscape of MS is not yet fully characterized, and scanty data are available (Table 3).

**Table 3:** Mutational landscape in myeloid sarcoma

Mutated genes and cytogenetic alterations	Rates (%)	References
*NPM1*	15–54	[63, 85, 88, 90]
*NRAS*	11–31	[85, 90, 87–]
*IDH2*	11–31	[86, 90, 92]
*DNMT3A*	8–28	[86, 88, 92]
*TET2*	17–22	[86, 88]
*TP53*	8–22	[88, 92]
*FLT3*-TKD	17	[86]
*IDH1*	15	[92]
*KRAS*	11–15	[86, 88, 90]
*PTPN11*	11–15	[86, 92]
*FLT3*-ITD	6–15	[85, 86, 90]
*cKIT*	14–15	[88, 90]
*CBL*	11	[86, 88]
*RUNX1*	7–11	[86, 88]
*RUNX1*::*RUNX1T1*	2–23	[13, 88, 90]
*CBFB::MYH11*	9–17	[13, 86, 88]
Trisomy 8	11–15	[13, 88, 90]
Monosomy 7	8–11	[13, 90]
*MLL* rearrangement	7–11	[13, 88, 90]
del(5q)	5–8	[13, 90]

*FLT3-*ITD mutations were the first mutation to be identified in MS cells, in up to 15% of cases [85, 86]. Limited data support a *FLT3*-TKD mutation rate of 17%, bringing the total rate of *FLT3* mutations in MS to about 25–30%, which is quietly similar to those reported in AML [86]. However, a small recent study revealed a lower frequency of *FLT3* mutations in the context of MS (1/16 cases, 6%) [87]. Considering different series together, *NPM1* mutations were detected in 20–50% of cases [64, 86–88]. Recently, *NPM1* mutated MS (*N* = 43) and AML (*n* = 106) were compared [89]; MS had more frequent cytogenetic abnormalities including complex karyotype and was enriched in mutations of genes involved in histone modification, including *ASXL1*. Conversely, *NPM1* mutated AML, which displayed a better overall survival compared to *NPM1* mutated MS, harboured a higher average number of gene mutations including *PTPN11*, *DNMT3A*, and *IDH1* [89].

Other reports demonstrated that mutations in the RAS pathway, comprising *KRAS*, *NRAS*, *BRAF*, *PTPN11*, and *CBL*, are common in MS with a rate of 30–85%, mostly *KRAS*/*NRAS* which accounted for 70% [86, 88, 90, 91]. Information on *IDH1/2* mutations in patients with MS is scanty. In a small case series on isolated MS, *IDH* mutations were reported in 6/14 (42%) patients (four *IDH2* and two *IDH1*) [92], whereas a study reported a *IDH2* mutation in 1 of 7 patients with MS using paired MS and bone marrow samples [93]. Recently, Ball et al. documented *IDH1* mutations in 5/19 (26%) patients, whereas 2/19 (11%) harboured *IDH2* mutation in MS.

Highlighting the different subsets of MS, Pastoret et al. [88] evaluated the mutational status of MS arising in two groups: isolated/concomitant with AML and MS secondary to MPN/MDS. Mutations in *DNMT3A*, *RUNX1*, *TP53*, *IDH2*, *NPM1*, *NRAS*, *KIT*, and *TET2* were found in the first group, whereas *SF3B1* and *SRSF2* mutations were found predominantly in the latter. Moreover, they compared genetics of MS to their marrow counterparts; 9/14 patients (64%) tested were found to harbour between 1 and 5 mutations. Of note, 2/9 patients had discordant results for *DNMT3A*, *RUNX1*, and *TP53* (documented in MS but not in non-infiltrated bone marrow). In another study by Kashofer et al. [86] including 18 patients with MS (11 synchronous with AML and 7 isolated), *NPM1*, *NRAS*, and *DNMT3A* mutations were the most frequent. Of note, neither *FLT3*-ITD nor *IDH2* mutations were reported in isolated MS cases and paired MS/bone marrow analysis in synchronous cases documented the same mutational landscape. The latter suggests that the risk profile obtained from leukemic BM might be sufficient and additional analysis of MS specimens is not strictly necessary. Overall, this result supports the conclusions of Ganzel et al. [24] who failed to observe, in a large retrospective analysis of 11 clinical trials from 1980 to 2008, a prognostic effect of extramedullary manifestations in AML patients treated with chemotherapy, concluding that additional biopsy of suspected MS sites might be not necessary in case AML diagnosed by BM analysis. However, intrinsic limitations of the latter study and low number of cases included in the previous reports are far from being adequate to clearly address this specific issue.

## Prognostic implications

The prognosis in patients with both isolated and synchronous MS is controversial being largely dependent on tumour site, timing of presentation, genetics, and treatment strategies. Concerning site of involvement, one study reported differences with better outcomes for isolated MS involving the pelvis/genitourinary organs, eyes/gonads, and GI mucosa compared with disease localization in primary soft tissues, lymphatic/hematopoietic organs, or CNS [23].

Although historical assessments on the independent prognostic effect of MS supported inferior outcomes [94, 95], later studies have reported better survival with isolated MS when compared with both AML without MS and synchronous AML [22, 96], also including paediatric patients [97]; other large retrospective studies did not show a clear prognostic impact [13, 24, 33]. A better outcome of isolated MS when compared to pure AML or synchronous MS/AML was also reported in setting post allo-HSCT relapse [19]. A history of MDS or MPN seems to have a negative prognostic impact on survival in patients with MS [22, 33].

## Therapeutic approaches

Treatment strategies are limited because of the rarity of the disease and lack of randomized clinical trials. Therapeutic choice is influenced by the different subsets including isolated MS versus synchronous MS, newly diagnosed, or relapsed, also including post allo-HSCT setting. Treatment with systemic AML protocols is the most reasonable approach as virtually all patients with MS eventually develop AML. Different therapeutic strategies are also dictated by size and location of MS (skin, CNS, or others) and patient’s specific factors as age, performance status (PS), and comorbidities. Considering all these factors, variable modalities of treatment can be utilized, including local therapy, chemotherapy, hematopoietic stem cell transplantation, targeted therapies, and immunotherapies.

### Local therapies

Local therapy includes either surgery or radiotherapy (RT). Up to 70% of patients with MS may have local symptoms; accordingly, local therapy provides an expedient palliation. In this regard, few reports support the use of surgery before the initiation of systemic treatment. Conversely, in some cases in which the diagnosis is difficult, surgical excision biopsy may be useful. Overall, an aggressive surgical approach is not supported since MS appears to be exquisitely sensitive to ionizing radiation. Involved field RT should be considered for patients with isolated MS and is recommended for all patients with MS refractory to systemic therapy[98]. Concerning radiation dose, one of the first study on 23 cases of MS reported a dose-response relationship with RT and most benefit from treatment with more than 10 Gy [99]. More recent studies suggest that a range of doses from 10 to 30 Gy over 1–3 weeks is highly effective [100, 101].

Radiotherapy in addition to chemotherapy in MS was evaluated in limited trials, and although one trial suggested a potential survival benefit for the addition of radiotherapy to chemotherapy [21], a large retrospective series of 71 patients (including studies from 1990 to 2014) showed no benefit from combination therapy [102]. Overall, recently published Guidelines From the International Lymphoma Radiation Oncology Group recommended RT, mostly using a low-dose regimen of 24 Gy in 12 fractions with conventional techniques, in the following scenarios: (i) for patients with isolated MS and inadequate response to chemotherapy, (ii) with isolated recurrence after allo-HSCT, and (iii) for palliation of symptomatic vital structure compression [103].

### Systemic therapies

The role of induction chemotherapy in MS is supported by several studies even in isolated cases, given that most (71–100%) patients treated with localized therapies (surgery and/or radiotherapy) progress to AML at a median of 4–6 months [21, 50, 53, 104, 105]. In isolated MS, systemic chemotherapy also has been shown to decrease progression to AML and increase overall survival. In addition, time to progression to AML was longer in those treated with systemic chemotherapy as opposed to local radiotherapy or surgery. These observations support the NCCN recommendation that patients with isolated MS (as those with synchronous MS), if eligible, must be treated with systemic therapy as per AML [98]. Conversely, the latest European LeukemiaNet (ELN) guidelines offer no specific recommendations [106].

No MS-specific treatment regimens have been adopted; intensive therapy-eligible patients are classically treated with anthracycline and cytarabine-based regimens [21, 53, 94]. Controlled clinical trials including and/or specific to MS patients are missing, making the superiority of one intensive regimen over the others unknown.

For intensive therapy-ineligible patients, hypomethylating agents (HMA) as azacitidine (5-azacytidine) [107–111] and decitabine (5-aza-2′-deoxycytidine) [112–116] were reported to induce clinical remissions, yet at varying degrees, in few reports. Overall, they were used in less than 20 cases including treatment-naïve and relapse/refractory MS.

Almost all the studies cited above describing the treatment of MS were performed prior to the availability of approved targeted therapies for AML, which have broadened the options and potentially improved the outcomes. In particular, since 2017, several therapies have received regulatory approval, alone and/or in combination with chemotherapy, including CPX-351 (a liposomal formulation of cytarabine and daunorubicin at a fixed 5:1 molar ratio); kinase inhibitors such as sorafenib, midostaurin and gilteritinib, ivosidenib, and enasidenib (IDH1 and IDH2 inhibitors, respectively); BCL2 inhibitor venetoclax; and glasdegib, an inhibitor of the transmembrane protein smoothened (SMO) involved in the Hedgehog signalling pathway [117]. Among all these therapies, only few data concerning efficacy in MS are reported, mainly in case reports.

Concerning the use of sorafenib for the treatment of MS, there are results from a small phase II study including 26 refractory AML cases with CNS involvement with 8 of them having *FLT3-*ITD mutation. After 8 weeks of treatment with sorafenib in combination with conventional chemotherapy, 21 patients achieved complete remission (CR), 2 achieved partial response, and 3 were refractory, resulting in an overall CR rate of 80.8% and an overall response rate of 88.5%. Of note, the 2-year event-free survival and OS rates were 75.0% and 76.9%, respectively [118]. Moreover, at least five case reports demonstrating efficacy of gilteritinib in the subset of *FLT3* mutated MS were published [119–124].

As above reported, *IDH1*/*IDH2* mutations have been described in MS; some published data support the use of enasidenib or ivosidenib in these patients. In this regards, in a retrospective series of 58 MS cases, treatment with ivosidenib led to a complete response (CR) in 2 of 3 patients with *IDH1* mutations. One patient with an *IDH2* mutation was treated with enasidenib for MS and experienced CR. The median duration of response was 15 months (range 7–18 months), with an estimated median OS of 26.6 months in patients responding to IDH1 and IDH2 inhibitors [87].

Moreover, few other cases highlighting venetoclax efficacy in the context of MS were described [70, 125–127]. In this regard, the diffusion of venetoclax through the cerebrospinal membrane might be advantageous for treating CNS involvement [128].

### Allogeneic hematopoietic stem cell transplantation

Nowadays, there are no controlled prospective clinical trials evaluating the role of allo-HSCT as post-remission therapy in patients with isolated MS or concomitant AML and MS. Due to its potential immunological anti-leukemic effect, it has been hypothesized that allo-HSCT should be always used in first remission to overcome the potential poor prognostic impact of MS [94, 95]. On the contrary, isolated MS relapses, usually hiding and anticipating a systemic relapse, are relatively common following allo-HSCT indicating a relative lack of graft versus leukaemia effect [32]. In this regard, in different dated studies, reduced intensity conditioning (RIC) regimens, T cell depleted grafts, or non-total body irradiation (TBI)-based conditioning regimens have been associated with higher rates of MS relapse and may reduce the effectiveness of allo-HSCT in AML with MS [26, 129–131]. Overall, contemporary data supporting allo-HSCT in first remission in all the patients with MS are lacking.

More recently, the outcome of allo-HSCT was evaluated in three large retrospective series involving mostly synchronous AML/MS. In a retrospective analysis of 51 patients with MS (with only 12% as isolated MS), the 5-year OS was 47% with a median follow-up of 33 months [132]; similar results were reported in a study from a large Japan cohort of 503 consecutive adult AML patients (median age, 44 years; range, 15–73 years) who received allo-HSCT, including 44 patients with MS (8.7%). Overall, comparable survival was reported in patients with and without MS (5-year OS was 47% and 44%, respectively) [133]. In the latest and largest study, using data from the Center for International Blood and Marrow Transplant Research including 310 centres and 44 different countries, the presence of MS at any time before allo-HSCT did not adversely affect the outcomes in 813 patients when compared with a cohort of AML patients without MS (*N* = 8983) [134]. Moreover, the presence of MS did not affect OS, leukaemia-free survival, treatment-related mortality, or risk of relapse, also in multivariable analysis, and the outcome was not influenced by the location, timing (concomitant or synchronous MS), or intensity of conditioning regimen [134]. The authors also tested for any interaction between the presence of concomitant MS and the intensity of the conditioning regimen on the risk of relapse. Among those with extramedullary disease, the rate of patients underwent myeloablative with TBI, myeloablative without TBI and non-myeloablative conditioning regimens were 47%, 35%, and 18%, respectively. Specifically, they did not identify any interaction between myeloablative (including TBI) and non-myeloablative conditioning on the risk of relapse. In particular, after myeloablative conditioning regimen, the relative risk of relapse was 1.09 (95% CI, 0.95–1.24; *P* = 0.21) and for reduced intensity conditioning was 0.89 (95% CI, 0.70–1.14; *P* = 0.36) [134]. More recently, although without a sub-analysis for adult AML patients with and without extramedullary involvement, TBI (12 Gy) plus fludarabine versus busulfan plus fludarabine as a myeloablative conditioning before allo-HSCT in patients with AML were comparable in relation to efficacy and safety in both first and second remissions [135]. Similarly, a recent large retrospective study including paediatric AML patients treated with and without TBI in the context of myeloablative conditioning regimens failed to demonstrate the clear advantage of TBI in terms of overall and leukaemia free survival [136].

More specifically, in the setting of isolated MS, consolidation with allo-HSCT has not been adequately studied, mainly due to low number of cases and heterogeneity of presentation [13, 132]. In the light of these observation, although limited and retrospective, the consolidation therapy for MS (with and without concomitant AML) should follow the same approach used for AML; accordingly, patients with higher risk (depending on cytogenetic and molecular profiles) should undergo allo-HSCT, hopefully in first remission, whereas consolidation chemotherapy should be reserved for patients with lower risk disease or patients who are unfit for allo-HSCT. Moreover, allo-HSCT should be considered in all cases of relapsed/refractory MS.

Finally, lacking clear evidences, the choice of conditioning regimen should be based on patients’ medical history and comorbidities, availability of TBI, and the experience of the individual centre.

## Conclusions

MS is a rare entity among myeloid neoplasms, probably underestimated, which can occur at any site with and without a bone marrow involvement. The diagnosis, particularly in isolated cases, may be difficult since MS can mimic other diseases, particularly other myeloid or lymphoproliferative neoplasms. Therefore, immunohistochemistry is mandatory for diagnosis and an extensive antibody panel should be performed, preferably on excision or core biopsies. Although to date rarely performed, cytogenetic and molecular analysis on MS biopsy could be useful to risk stratify patients and guide treatment strategies. A bone marrow evaluation, including immunophenotyping, cytogenetic, and molecular analysis, is mandatory in all the cases to exclude the possibility of a concomitant AML. Conversely, a biopsy of suspected MS might be not necessary in AML cases diagnosed by bone marrow analysis.

No consensus management guidelines are available. Since isolated MS is predictive of intramedullary disease, induction systemic therapies (chemotherapy alone or combined with target drugs) appear to provide a survival benefit over local treatments (mainly radiotherapy). Consolidation treatment remains controversial, and radiotherapy, systemic therapies, and/or allo-HSCT should be adopted depending on extent of involvement, risk profile, and performance status of individual patient. Relapsed MS should be treated as relapsed AML, including the use of recently approved agents, if indicated.

As described above, some targeted drugs have shown efficacy in the treatment of MS; however, in almost all cases, they have been used in refractory/relapsed MS setting, after multiple lines of conventional therapies. This can lead to drug resistance or clonal evolution, reducing the effectiveness of treatment. Accordingly, the earlier use of novel agents could be beneficial for patients with MS.

Further studies and inclusion of patients with MS in large multicentre prospective clinical trials, to better identify the best clinical management, are needed.

## Data Availability

Not applicable

## Declarations

### Disclaimer

The funders had no role in the design of the study; in the collection, analyses, and interpretation of data; and in the writing of the present manuscript.
